# (*E*)-2-(4-*tert*-But­ylphen­yl)-2-cyano-1-(3-methyl-1-vinyl-1*H*-pyrazol-5-yl)vinyl 2,2-dimethyl­propano­ate

**DOI:** 10.1107/S1600536811052007

**Published:** 2011-12-10

**Authors:** Guiqiu Yang, Yang Wang, Haibo Yu

**Affiliations:** a Shenyang University of Chemical Technology, Shenyang 110142, People’s Republic of China; b Agrochemicals Division, Shenyang Research Institute of Chemical Industry, Shenyang 110021, People’s Republic of China

## Abstract

In the title compound, C_24_H_29_N_3_O_2_, the dihedral angle between the benzene and pyrazole rings is 80.55 (7)°. The mol­ecule contains an acrylonitrile moiety and exists in an *E* conformation. Bioassay tests showed that the title compound exhibited higher acaricidal activity than its *Z* isomer.

## Related literature

For background to acrylonitrile compounds, see: Boedec *et al.* (2008 ▶); Napolitano *et al.* (2001 ▶); Reggio *et al.* (1998 ▶). For further synthetic details, see: Kenzo *et al.* (2006 ▶); Yang *et al.* (2009 ▶).
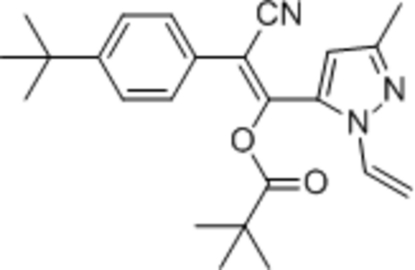


## Experimental

### 

#### Crystal data

C_24_H_29_N_3_O_2_
*M* *_r_* = 391.50
Orthorhombic, 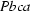

*a* = 12.0056 (16) Å
*b* = 19.283 (3) Å
*c* = 20.183 (3) Å
*V* = 4672.5 (11) Å^3^
*Z* = 8
Mo *K*α radiation
μ = 0.07 mm^−1^
*T* = 296 K
0.38 × 0.36 × 0.32 mm

#### Data collection

Bruker SMART CCD diffractometer
Absorption correction: multi-scan (*SADABS*; Bruker, 2001 ▶) *T*_min_ = 0.973, *T*_max_ = 0.978
22480 measured reflections
4118 independent reflections
2871 reflections with *I* > 2σ(*I*)
*R*_int_ = 0.033

#### Refinement

*R*[*F* ^2^ > 2σ(*F* ^2^)] = 0.048
*wR*(*F* ^2^) = 0.148
*S* = 1.05
4118 reflections
269 parameters
H-atom parameters constrained
Δρ_max_ = 0.31 e Å^−3^
Δρ_min_ = −0.20 e Å^−3^

### 

Data collection: *SMART* (Bruker, 2001 ▶); cell refinement: *SAINT* (Bruker, 2001 ▶); data reduction: *SAINT*; program(s) used to solve structure: *SHELXS97* (Sheldrick, 2008 ▶); program(s) used to refine structure: *SHELXL97* (Sheldrick, 2008 ▶); molecular graphics: *SHELXTL* (Sheldrick, 2008 ▶); software used to prepare material for publication: *SHELXTL*.

## Supplementary Material

Crystal structure: contains datablock(s) I, global. DOI: 10.1107/S1600536811052007/hb6506sup1.cif

Structure factors: contains datablock(s) I. DOI: 10.1107/S1600536811052007/hb6506Isup2.hkl

Supplementary material file. DOI: 10.1107/S1600536811052007/hb6506Isup3.cml

Additional supplementary materials:  crystallographic information; 3D view; checkCIF report

## Figures and Tables

**Fig. 1.**
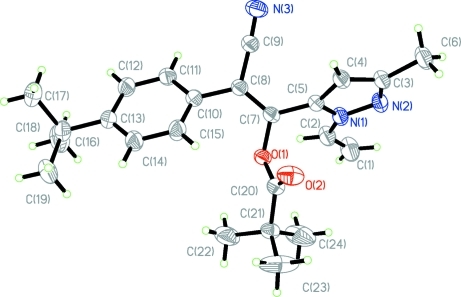
The molecular structure of (I), with 30% probability displacement ellipsoids.

**Fig. 2.**
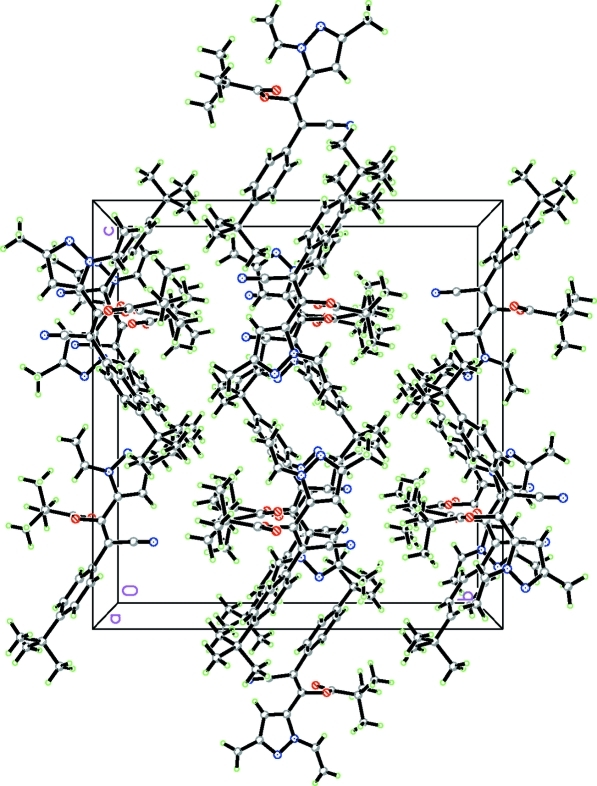
Crystal packing of (I).

## supplementary crystallographic information

### Comment

Acrylonitrile compounds display a broad range of biological, medical and pharmacological properties (Napolitano *et al.*, 2001, Boedec *et al.*, 2008). There is a double bond in the molecule of the acrylonitrile compounds, and both geometric isomers referred to as the E– and *Z*-isomer can be present. The bioactivities of them often differ from each other (Reggio *et al.*,1998). In the process of preparation of the title compound, its geometric isomer product was also afforded, which showed obviously different acaricidal activity with the title compound. The bioassay tests showed that the title compound exhibited higher acaricidal activity than its isomer.

In order to confirm the geometry configuration, we report the crystal structure of the title compound (I) in this paper. The molecular structure of (I) is shown in Fig. 1. The the benzene and pyrazole rings in each of the ligands are not coplanar, the dihedral angle formed by the least-squares planes of the benzene and pyrazole rings being equal to 80.55 (7)°. The C(9)—C(8)—C(10)—C(11), O(1)—C(7)—C(8)—C(9), C(5)—C(7)—C(8)—C(9) and C(20)—O(1)—C(7)—C(5) torsion angles are 38.7 (3), -168.00 (2), 5.8 (3) and 66.1 (2)°, respectively. The crystal packing of (I) shows in Fig. 2. No significant interactions, such as hydrogen bonds or pi-pi stacking, are observed in (I).

### Experimental

The title compound was synthesized by 2-(4-(*tert*-butyl) phenyl)-3-(4-chloro-1-ethyl-3-methyl-1*H*-pyrazol-5-yl) -3-hydroxyacrylonitrile with pivaloyl chloride in THF (Kenzo *et al.*, 2006, Yang *et al.*, 2009). The crude products were purified by silica-gel column chromatography and then grown from heptane to afford colorless single crystals suitable for X-ray diffraction. To the mixture of 2-(4-(*tert*-butyl)phenyl)-3-(3-methyl-1-vinyl- 1*H*-pyrazol-5-yl)-3-hydroxyacrylonitrile (0.61 g, 2.0 mmol) and triethyl amine (0.24 g, 2.4 mmol) in THF (10 ml), pivaloyl chloride (0.29 g, 2.4 mmol) was added dropwise at roomtemperature and reacted for 1 h. After separation through silica gel column chromatography (fluent: ethyl acetate/petroleum ether=1/10), The title product compound was gained as a white solid (0.59 g, 75%).

Anal. Calcd for C_24_H_29_N_3_O_2_: C, 73.63; H, 7.47; N, 10.73. Found: C, 73.67; H, 7.50; N, 10.75. ^1^H NMR(DMSO): 1.14 (s, 9H, CO (CH_3_)_3_), 1.33 (s, 9H, Ph-(CH_3_)_3_), 2.34 (s, 3H, CH_3_), 4.92 (d, 1H, CH), 5.79 (d, 1H, CH), 6.56 (s, 1H, py), 7.07 (dd, 1H, CH), 7.45 (d, 2H, Ph), 7.48 (d, 2H, Ph).

### Refinement

Although all H atoms were visible in difference maps, they were finally placed in geometrically calculated positions, with C—H distances in the range 0.93–0.96 Å, and included in the final refinement in the riding model approximation, with *U*_iso_(H) = 1.2*U*_eq_(C) and *U*_iso_(H) = 1.5*U*_eq_(C).

### Crystal data

**Table:**

C_24_H_29_N_3_O_2_	*D*_x_ = 1.113 Mg m^−^^3^
*M**_r_* = 391.50	Mo *K*α radiation, λ = 0.71073 Å
Orthorhombic, *P**b**c**a*	Cell parameters from 4156 reflections
*a* = 12.0056 (16) Å	θ = 2.6–20.4°
*b* = 19.283 (3) Å	µ = 0.07 mm^−^^1^
*c* = 20.183 (3) Å	*T* = 296 K
*V* = 4672.5 (11) Å^3^	Block, colorless
*Z* = 8	0.38 × 0.36 × 0.32 mm
*F*(000) = 1680	

### Data collection

**Table:**

Bruker SMART CCD diffractometer	4118 independent reflections
Radiation source: fine-focus sealed tube	2871 reflections with *I* > 2σ(*I*)
graphite	*R*_int_ = 0.033
phi and ω scans	θ_max_ = 25.0°, θ_min_ = 2.0°
Absorption correction: multi-scan (*SADABS*; Bruker, 2001)	*h* = −14→12
*T*_min_ = 0.973, *T*_max_ = 0.978	*k* = −22→22
22480 measured reflections	*l* = −21→24

### Refinement

**Table:**

Refinement on *F*^2^	Secondary atom site location: difference Fourier map
Least-squares matrix: full	Hydrogen site location: inferred from neighbouring sites
*R*[*F*^2^ > 2σ(*F*^2^)] = 0.048	H-atom parameters constrained
*wR*(*F*^2^) = 0.148	*w* = 1/[σ^2^(*F*_o_^2^) + (0.0633*P*)^2^ + 1.5443*P*] where *P* = (*F*_o_^2^ + 2*F*_c_^2^)/3
*S* = 1.05	(Δ/σ)_max_ < 0.001
4118 reflections	Δρ_max_ = 0.31 e Å^−^^3^
269 parameters	Δρ_min_ = −0.20 e Å^−^^3^
0 restraints	Extinction correction: *SHELXL97* (Sheldrick, 2008), Fc^*^=kFc[1+0.001xFc^2^λ^3^/sin(2θ)]^-1/4^
Primary atom site location: structure-invariant direct methods	Extinction coefficient: 0.0021 (4)

### Special details

**Table:**

Geometry. All e.s.d.'s (except the e.s.d. in the dihedral angle between two l.s. planes) are estimated using the full covariance matrix. The cell e.s.d.'s are taken into account individually in the estimation of e.s.d.'s in distances, angles and torsion angles; correlations between e.s.d.'s in cell parameters are only used when they are defined by crystal symmetry. An approximate (isotropic) treatment of cell e.s.d.'s is used for estimating e.s.d.'s involving l.s. planes.
Refinement. Refinement of *F*^2^ against ALL reflections. The weighted *R*-factor *wR* and goodness of fit *S* are based on *F*^2^, conventional *R*-factors *R* are based on *F*, with *F* set to zero for negative *F*^2^. The threshold expression of *F*^2^ > σ(*F*^2^) is used only for calculating *R*-factors(gt) *etc*. and is not relevant to the choice of reflections for refinement. *R*-factors based on *F*^2^ are statistically about twice as large as those based on *F*, and *R*- factors based on ALL data will be even larger.

### Fractional atomic coordinates and isotropic or equivalent isotropic displacement parameters (Å^2^)

**Table:**

	*x*	*y*	*z*	*U*_iso_*/*U*_eq_	
O1	1.01105 (11)	0.08044 (6)	0.73974 (7)	0.0513 (4)	
O2	1.19100 (13)	0.05098 (8)	0.73607 (9)	0.0768 (5)	
N1	0.97347 (15)	−0.00910 (9)	0.85788 (8)	0.0567 (5)	
N2	1.00594 (16)	−0.05609 (9)	0.90444 (9)	0.0646 (5)	
N3	0.8306 (2)	−0.13197 (10)	0.68953 (12)	0.0864 (7)	
C1	0.8888 (3)	0.07058 (15)	0.93404 (14)	0.0988 (10)	
H1A	0.9211	0.0479	0.9699	0.119*	
H1B	0.8437	0.1091	0.9410	0.119*	
C2	0.9067 (2)	0.04860 (12)	0.87455 (12)	0.0699 (7)	
H2	0.8729	0.0726	0.8400	0.084*	
C3	1.05513 (19)	−0.10678 (11)	0.87092 (11)	0.0593 (6)	
C4	1.05432 (18)	−0.09274 (10)	0.80299 (11)	0.0576 (5)	
H4	1.0837	−0.1202	0.7694	0.069*	
C5	1.00186 (16)	−0.03077 (10)	0.79596 (10)	0.0500 (5)	
C6	1.1022 (2)	−0.16827 (13)	0.90619 (13)	0.0807 (8)	
H6A	1.1816	−0.1636	0.9094	0.121*	
H6B	1.0844	−0.2097	0.8820	0.121*	
H6C	1.0709	−0.1710	0.9499	0.121*	
C7	0.97899 (16)	0.01082 (9)	0.73651 (9)	0.0485 (5)	
C8	0.92256 (16)	−0.01145 (10)	0.68363 (10)	0.0494 (5)	
C9	0.87266 (19)	−0.07898 (11)	0.68833 (11)	0.0601 (6)	
C10	0.90092 (17)	0.02826 (10)	0.62182 (10)	0.0512 (5)	
C11	0.79697 (18)	0.02586 (12)	0.59251 (11)	0.0616 (6)	
H11	0.7419	−0.0023	0.6105	0.074*	
C12	0.77404 (19)	0.06478 (12)	0.53681 (11)	0.0655 (6)	
H12	0.7033	0.0624	0.5182	0.079*	
C13	0.85333 (19)	0.10742 (11)	0.50771 (10)	0.0580 (5)	
C14	0.9582 (2)	0.10713 (13)	0.53610 (11)	0.0702 (7)	
H14	1.0142	0.1338	0.5172	0.084*	
C15	0.98194 (19)	0.06812 (13)	0.59201 (11)	0.0660 (6)	
H15	1.0535	0.0689	0.6096	0.079*	
C16	0.8234 (2)	0.15167 (13)	0.44748 (11)	0.0702 (7)	
C17	0.7940 (3)	0.10424 (16)	0.38919 (13)	0.1009 (10)	
H17A	0.8573	0.0761	0.3781	0.151*	
H17B	0.7735	0.1320	0.3516	0.151*	
H17C	0.7327	0.0749	0.4013	0.151*	
C18	0.7231 (3)	0.19699 (17)	0.46499 (16)	0.1115 (11)	
H18A	0.6607	0.1680	0.4758	0.167*	
H18B	0.7045	0.2258	0.4278	0.167*	
H18C	0.7411	0.2257	0.5024	0.167*	
C19	0.9185 (3)	0.19962 (16)	0.42640 (15)	0.1063 (11)	
H19A	0.9387	0.2291	0.4628	0.160*	
H19B	0.8949	0.2276	0.3896	0.160*	
H19C	0.9817	0.1723	0.4135	0.160*	
C20	1.12292 (17)	0.09525 (10)	0.74372 (10)	0.0518 (5)	
C21	1.14310 (17)	0.17108 (10)	0.75803 (11)	0.0556 (5)	
C22	1.0765 (3)	0.21582 (14)	0.7115 (2)	0.1206 (13)	
H22A	1.0931	0.2637	0.7197	0.181*	
H22B	0.9984	0.2078	0.7186	0.181*	
H22C	1.0953	0.2045	0.6666	0.181*	
C23	1.2642 (2)	0.18635 (16)	0.7505 (2)	0.1382 (17)	
H23A	1.2859	0.1792	0.7052	0.207*	
H23B	1.3064	0.1560	0.7787	0.207*	
H23C	1.2783	0.2337	0.7627	0.207*	
C24	1.1051 (4)	0.18545 (16)	0.82782 (17)	0.1350 (16)	
H24A	1.1466	0.1570	0.8581	0.202*	
H24B	1.0272	0.1750	0.8317	0.202*	
H24C	1.1173	0.2335	0.8381	0.202*	

### Atomic displacement parameters (Å^2^)

**Table:**

	*U*^11^	*U*^22^	*U*^33^	*U*^12^	*U*^13^	*U*^23^
O1	0.0501 (8)	0.0407 (7)	0.0630 (9)	−0.0020 (6)	−0.0045 (6)	0.0059 (6)
O2	0.0561 (9)	0.0525 (9)	0.1218 (14)	0.0054 (8)	0.0108 (9)	−0.0098 (9)
N1	0.0695 (12)	0.0507 (10)	0.0500 (10)	0.0061 (9)	−0.0031 (8)	0.0059 (8)
N2	0.0793 (13)	0.0600 (11)	0.0544 (10)	0.0039 (10)	−0.0052 (9)	0.0125 (9)
N3	0.0908 (16)	0.0511 (12)	0.1174 (18)	−0.0115 (11)	−0.0258 (13)	0.0096 (12)
C1	0.145 (3)	0.0825 (19)	0.0688 (18)	0.0329 (19)	0.0055 (17)	0.0000 (14)
C2	0.0892 (18)	0.0611 (14)	0.0593 (14)	0.0129 (13)	0.0022 (12)	0.0055 (11)
C3	0.0642 (14)	0.0517 (12)	0.0620 (13)	0.0014 (11)	−0.0064 (11)	0.0130 (10)
C4	0.0654 (13)	0.0489 (12)	0.0587 (13)	0.0042 (10)	−0.0022 (10)	0.0059 (10)
C5	0.0534 (12)	0.0448 (11)	0.0519 (11)	−0.0020 (9)	−0.0034 (9)	0.0049 (9)
C6	0.0979 (19)	0.0665 (15)	0.0776 (16)	0.0142 (14)	−0.0088 (14)	0.0238 (13)
C7	0.0490 (11)	0.0411 (10)	0.0554 (12)	0.0007 (9)	0.0007 (9)	0.0054 (9)
C8	0.0504 (11)	0.0442 (11)	0.0537 (12)	0.0005 (9)	−0.0020 (9)	0.0029 (9)
C9	0.0648 (14)	0.0477 (13)	0.0676 (14)	0.0004 (11)	−0.0119 (11)	0.0053 (10)
C10	0.0547 (12)	0.0468 (11)	0.0522 (11)	0.0009 (9)	−0.0039 (9)	0.0014 (9)
C11	0.0564 (13)	0.0635 (13)	0.0649 (13)	−0.0116 (11)	−0.0075 (10)	0.0085 (11)
C12	0.0586 (14)	0.0743 (15)	0.0637 (14)	−0.0059 (12)	−0.0145 (11)	0.0092 (12)
C13	0.0682 (14)	0.0568 (12)	0.0491 (12)	−0.0019 (11)	−0.0072 (10)	0.0013 (10)
C14	0.0679 (15)	0.0834 (17)	0.0593 (14)	−0.0164 (13)	−0.0032 (11)	0.0163 (12)
C15	0.0520 (13)	0.0869 (17)	0.0591 (13)	−0.0097 (12)	−0.0073 (10)	0.0130 (12)
C16	0.0887 (18)	0.0700 (15)	0.0520 (13)	0.0016 (13)	−0.0084 (12)	0.0084 (11)
C17	0.139 (3)	0.106 (2)	0.0578 (15)	−0.004 (2)	−0.0199 (16)	0.0021 (15)
C18	0.133 (3)	0.107 (2)	0.095 (2)	0.043 (2)	−0.0074 (19)	0.0255 (18)
C19	0.132 (3)	0.103 (2)	0.083 (2)	−0.021 (2)	−0.0145 (18)	0.0406 (17)
C20	0.0504 (12)	0.0487 (11)	0.0562 (12)	−0.0004 (10)	0.0018 (9)	0.0025 (9)
C21	0.0500 (12)	0.0434 (11)	0.0734 (14)	−0.0003 (9)	0.0010 (10)	−0.0006 (10)
C22	0.124 (3)	0.0591 (16)	0.179 (3)	−0.0163 (17)	−0.050 (2)	0.0348 (19)
C23	0.0617 (18)	0.0661 (17)	0.287 (5)	−0.0136 (14)	0.013 (2)	−0.039 (3)
C24	0.229 (5)	0.0684 (18)	0.107 (3)	−0.036 (2)	0.047 (3)	−0.0325 (18)

### Geometric parameters (Å, °)

**Table:**

O1—C20	1.375 (2)	C13—C16	1.528 (3)
O1—C7	1.398 (2)	C14—C15	1.386 (3)
O2—C20	1.192 (2)	C14—H14	0.9300
N1—C5	1.361 (2)	C15—H15	0.9300
N1—N2	1.362 (2)	C16—C18	1.529 (4)
N1—C2	1.412 (3)	C16—C19	1.530 (4)
N2—C3	1.327 (3)	C16—C17	1.531 (3)
N3—C9	1.140 (3)	C17—H17A	0.9600
C1—C2	1.291 (3)	C17—H17B	0.9600
C1—H1A	0.9300	C17—H17C	0.9600
C1—H1B	0.9300	C18—H18A	0.9600
C2—H2	0.9300	C18—H18B	0.9600
C3—C4	1.398 (3)	C18—H18C	0.9600
C3—C6	1.494 (3)	C19—H19A	0.9600
C4—C5	1.358 (3)	C19—H19B	0.9600
C4—H4	0.9300	C19—H19C	0.9600
C5—C7	1.469 (3)	C20—C21	1.510 (3)
C6—H6A	0.9600	C21—C23	1.491 (4)
C6—H6B	0.9600	C21—C22	1.505 (4)
C6—H6C	0.9600	C21—C24	1.506 (4)
C7—C8	1.335 (3)	C22—H22A	0.9600
C8—C9	1.436 (3)	C22—H22B	0.9600
C8—C10	1.487 (3)	C22—H22C	0.9600
C10—C15	1.378 (3)	C23—H23A	0.9600
C10—C11	1.382 (3)	C23—H23B	0.9600
C11—C12	1.379 (3)	C23—H23C	0.9600
C11—H11	0.9300	C24—H24A	0.9600
C12—C13	1.388 (3)	C24—H24B	0.9600
C12—H12	0.9300	C24—H24C	0.9600
C13—C14	1.383 (3)		
			
C20—O1—C7	118.09 (15)	C13—C16—C18	108.7 (2)
C5—N1—N2	110.95 (17)	C13—C16—C19	112.5 (2)
C5—N1—C2	127.07 (18)	C18—C16—C19	107.9 (2)
N2—N1—C2	121.49 (18)	C13—C16—C17	109.4 (2)
C3—N2—N1	105.40 (17)	C18—C16—C17	109.7 (2)
C2—C1—H1A	120.0	C19—C16—C17	108.6 (2)
C2—C1—H1B	120.0	C16—C17—H17A	109.5
H1A—C1—H1B	120.0	C16—C17—H17B	109.5
C1—C2—N1	125.1 (2)	H17A—C17—H17B	109.5
C1—C2—H2	117.5	C16—C17—H17C	109.5
N1—C2—H2	117.5	H17A—C17—H17C	109.5
N2—C3—C4	110.75 (18)	H17B—C17—H17C	109.5
N2—C3—C6	120.7 (2)	C16—C18—H18A	109.5
C4—C3—C6	128.6 (2)	C16—C18—H18B	109.5
C5—C4—C3	106.02 (19)	H18A—C18—H18B	109.5
C5—C4—H4	127.0	C16—C18—H18C	109.5
C3—C4—H4	127.0	H18A—C18—H18C	109.5
C4—C5—N1	106.87 (17)	H18B—C18—H18C	109.5
C4—C5—C7	130.71 (19)	C16—C19—H19A	109.5
N1—C5—C7	122.39 (17)	C16—C19—H19B	109.5
C3—C6—H6A	109.5	H19A—C19—H19B	109.5
C3—C6—H6B	109.5	C16—C19—H19C	109.5
H6A—C6—H6B	109.5	H19A—C19—H19C	109.5
C3—C6—H6C	109.5	H19B—C19—H19C	109.5
H6A—C6—H6C	109.5	O2—C20—O1	120.89 (18)
H6B—C6—H6C	109.5	O2—C20—C21	127.47 (19)
C8—C7—O1	119.07 (17)	O1—C20—C21	111.64 (17)
C8—C7—C5	124.90 (18)	C23—C21—C22	110.0 (3)
O1—C7—C5	115.76 (16)	C23—C21—C24	110.8 (3)
C7—C8—C9	116.77 (18)	C22—C21—C24	108.5 (3)
C7—C8—C10	126.43 (18)	C23—C21—C20	109.14 (19)
C9—C8—C10	116.71 (17)	C22—C21—C20	110.5 (2)
N3—C9—C8	177.0 (2)	C24—C21—C20	107.96 (19)
C15—C10—C11	117.98 (19)	C21—C22—H22A	109.5
C15—C10—C8	122.03 (18)	C21—C22—H22B	109.5
C11—C10—C8	119.99 (18)	H22A—C22—H22B	109.5
C12—C11—C10	120.7 (2)	C21—C22—H22C	109.5
C12—C11—H11	119.6	H22A—C22—H22C	109.5
C10—C11—H11	119.6	H22B—C22—H22C	109.5
C11—C12—C13	122.0 (2)	C21—C23—H23A	109.5
C11—C12—H12	119.0	C21—C23—H23B	109.5
C13—C12—H12	119.0	H23A—C23—H23B	109.5
C14—C13—C12	116.5 (2)	C21—C23—H23C	109.5
C14—C13—C16	123.1 (2)	H23A—C23—H23C	109.5
C12—C13—C16	120.4 (2)	H23B—C23—H23C	109.5
C13—C14—C15	121.8 (2)	C21—C24—H24A	109.5
C13—C14—H14	119.1	C21—C24—H24B	109.5
C15—C14—H14	119.1	H24A—C24—H24B	109.5
C10—C15—C14	120.9 (2)	C21—C24—H24C	109.5
C10—C15—H15	119.6	H24A—C24—H24C	109.5
C14—C15—H15	119.6	H24B—C24—H24C	109.5
			
C5—N1—N2—C3	0.3 (2)	C9—C8—C10—C15	−141.2 (2)
C2—N1—N2—C3	172.7 (2)	C7—C8—C10—C11	−137.6 (2)
C5—N1—C2—C1	−174.3 (3)	C9—C8—C10—C11	38.7 (3)
N2—N1—C2—C1	14.6 (4)	C15—C10—C11—C12	−2.9 (3)
N1—N2—C3—C4	−0.1 (2)	C8—C10—C11—C12	177.1 (2)
N1—N2—C3—C6	179.9 (2)	C10—C11—C12—C13	0.3 (4)
N2—C3—C4—C5	−0.1 (3)	C11—C12—C13—C14	2.2 (4)
C6—C3—C4—C5	179.9 (2)	C11—C12—C13—C16	−177.8 (2)
C3—C4—C5—N1	0.3 (2)	C12—C13—C14—C15	−2.1 (4)
C3—C4—C5—C7	178.3 (2)	C16—C13—C14—C15	177.9 (2)
N2—N1—C5—C4	−0.4 (2)	C11—C10—C15—C14	3.0 (3)
C2—N1—C5—C4	−172.3 (2)	C8—C10—C15—C14	−177.0 (2)
N2—N1—C5—C7	−178.57 (18)	C13—C14—C15—C10	−0.5 (4)
C2—N1—C5—C7	9.5 (3)	C14—C13—C16—C18	−124.4 (3)
C20—O1—C7—C8	−119.5 (2)	C12—C13—C16—C18	55.6 (3)
C20—O1—C7—C5	66.1 (2)	C14—C13—C16—C19	−5.0 (3)
C4—C5—C7—C8	57.1 (3)	C12—C13—C16—C19	175.0 (2)
N1—C5—C7—C8	−125.2 (2)	C14—C13—C16—C17	115.8 (3)
C4—C5—C7—O1	−128.9 (2)	C12—C13—C16—C17	−64.1 (3)
N1—C5—C7—O1	48.7 (3)	C7—O1—C20—O2	9.5 (3)
O1—C7—C8—C9	−168.00 (18)	C7—O1—C20—C21	−170.71 (16)
C5—C7—C8—C9	5.8 (3)	O2—C20—C21—C23	9.3 (4)
O1—C7—C8—C10	8.3 (3)	O1—C20—C21—C23	−170.5 (2)
C5—C7—C8—C10	−177.90 (19)	O2—C20—C21—C22	130.3 (3)
C7—C8—C9—N3	170 (5)	O1—C20—C21—C22	−49.5 (3)
C10—C8—C9—N3	−7(5)	O2—C20—C21—C24	−111.2 (3)
C7—C8—C10—C15	42.4 (3)	O1—C20—C21—C24	69.0 (3)
